# Bioinformatics Analysis of Spike Proteins of Porcine Enteric Coronaviruses

**DOI:** 10.1155/2021/6689471

**Published:** 2021-07-01

**Authors:** Yan Jia, Jinshan Cao, Zhanyong Wei

**Affiliations:** ^1^Laboratory of Veterinary Pharmacology, College of Veterinary Medicine, Inner Mongolia Agricultural University, No. 306, Zhaowuda Road, Saihan District, 010018 Hohhot, China; ^2^Key Laboratory of Animal-Derived Food Safety of Henan Province, Zhengzhou 450002, China

## Abstract

This article is aimed at analyzing the structure and function of the spike (S) proteins of porcine enteric coronaviruses, including transmissible gastroenteritis virus (TGEV), porcine epidemic diarrhea virus (PEDV), porcine deltacoronavirus (PDCoV), and swine acute diarrhea syndrome coronavirus (SADS-CoV) by applying bioinformatics methods. The physical and chemical properties, hydrophilicity and hydrophobicity, transmembrane region, signal peptide, phosphorylation and glycosylation sites, epitope, functional domains, and motifs of S proteins of porcine enteric coronaviruses were predicted and analyzed through online software. The results showed that S proteins of TGEV, PEDV, SADS-CoV, and PDCoV all contained transmembrane regions and signal peptide. TGEV S protein contained 139 phosphorylation sites, 24 glycosylation sites, and 53 epitopes. PEDV S protein had 143 phosphorylation sites, 22 glycosylation sites, and 51 epitopes. SADS-CoV S protein had 109 phosphorylation sites, 20 glycosylation sites, and 43 epitopes. PDCoV S protein had 124 phosphorylation sites, 18 glycosylation sites, and 52 epitopes. Moreover, TGEV, PEDV, and PDCoV S proteins all contained two functional domains and two motifs, spike_rec_binding and corona_S2. The corona_S2 consisted of S2 subunit heptad repeat 1 (HR1) and S2 subunit heptad repeat 2 (HR2) region profiles. Additionally, SADS-CoV S protein was predicted to contain only one functional domain, the corona_S2. This analysis of the biological functions of porcine enteric coronavirus spike proteins can provide a theoretical basis for the design of antiviral drugs.

## 1. Introduction

The pathogenic coronaviruses including porcine transmissible gastroenteritis virus (TGEV), porcine epidemic diarrhea virus (PEDV), swine acute diarrhea syndrome coronavirus (SADS), and porcine deltacoronavirus (PDCoV) have been found to be able to infect the intestinal tract of pigs [1]. Porcine intestinal diseases caused by these viruses are widespread in the world, causing serious losses to the pig industry. These four viruses are collectively referred to as porcine enteric coronaviruses.

Porcine enteric coronaviruses belong to the enveloped single-stranded and positive-sense RNA viruses of the order Nidovirales, Coronaviridae, and Coronavirus subfamily. The subfamily of coronaviruses is further divided into 4 genera according to the differences in genome sequence, which are called *α*, *β*, *γ*, and *δ* coronavirus. TGEV, PEDV, and SADS-CoV are members of the *α* genus, while PDCoV belongs to the *δ* genus [2, 3]. The genomes of coronaviruses contain four structural proteins: nucleocapsid (N), spike (S), envelope (E), and membrane (M). The S glycoprotein forms an 18-23 nm tall spike on the surface of the coronavirus, which is a typical type I virus fusion membrane protein [4, 5]. It can specifically bind to host cell receptors and mediate the invasion of viruses into susceptible cells and then determine the tissue tropism and host range of the virus [6].

The length of coronavirus S protein will change after glycosylation modification, and the molecular weight will also change accordingly. S protein is a homotrimer, and each monomer is divided into two regions, S1 and S2. S1 protein has a spherical structure and contains two independent functional subdomains, S1 subunit N-terminal (S1-NTD) and S1 subunit C-terminal (S1-CTD). S1 protein contains the corresponding receptor binding domain (RBD) that can bind to the host cell membrane [7]. The S1 carboxy-terminal domain can make the virus close to the surface of the host cell. The carboxy-terminal of S2 constitutes the stem of the spinous protein, which is mainly a highly conserved spiral structure. S2 consists of two heptapeptide repeat regions (HR1 and HR2), a transmembrane region (TM), and a fusion peptide (FP) region. S2 participates in the fusion between viral and host cellular membranes, cytopathic changes, and virus replication, as well as virus assembly and release [8]. Coronavirus receptor is an important factor in determining its host range and tissue tropism. The research about the determination of the coronavirus receptor and the binding mechanism between the virus and the receptor is beneficial for the prevention of new viruses and the development of related therapeutic drugs.

Studies have shown that TGEV enters cells by binding to porcine aminopeptidase N (pAPN) on the target cell membrane and using sialic acid as a cobinding factor [9]. Previous studies have shown that pAPN has also been identified as a functional receptor for PEDV, but the results of later studies are controversial with previous reports [10, 11]. PDCoV can use APN of multiple species as functional receptors, which is the main source of its cross-species transmission [12]. However, SADS-CoV does not use APN as its invasion receptor [13].

This article analyzed and compared the biological functions of the S protein of these four porcine enteric coronaviruses using bioinformatics software. The physical and chemical properties, transmembrane region, signal peptides, functional domains, protein modifications, and antigenic epitopes of porcine enteric coronavirus S protein were analyzed through bioinformatics software. Analyzing the biological functions of the S protein is helpful to study the biological characteristics of porcine enteric coronaviruses and at the same time provides data for the modification of the spinous protein and the design of antiviral drug molecules.

## 2. Materials and Methods

### 2.1. Virus Information

TGEV H16 (GenBank ID: FJ755618), PEDV CV777 (GenBank ID:AF353511), PDCoV HNZK-04 (GenBank ID:MH708124), and SADS GDS04 (GenBank ID: MF167434) S gene nucleotide and amino acid sequences were downloaded from NCBI (National Center for Biotechnology Information) (Table 1).

### 2.2. Bioinformatics Software

The physical characteristics and general biological characteristics of the porcine intestinal coronavirus S protein were calculated by the ProtParam and ProtScale tools on the ExPASy server. Through TMHMM Server v.2.0, SignalP4.0, NetPhos 3.1 Server, and NetNGlyc 4.0 Server software, we predicted the transmembrane region (transmembrane helix (TMH)), signal peptide, phosphorylation site, and glycosylation site of S protein, respectively. At the same time, the amino acid sequences of the S protein of porcine enteric coronavirus were submitted to Predicting Antigenic Peptides, SMART, and PROSITE which were used to perform the prediction of the epitopes, functional domains, and motifs of each S protein sequence. Multisequence alignment of porcine enteric coronavirus S protein was analyzed by Clustal Omega (Table 2).

## 3. Results

### 3.1. Physical and Chemical Properties of Porcine Enteric Coronavirus Spike Proteins

Upload the amino acid sequences of porcine enteric coronavirus spike proteins to the ProtParam online software, respectively. TGEV H16 S protein encoded 1448 amino acids, and its molecular weight and isoelectric point were 159888.38 and 5.26, respectively. The protein contained 126 negatively charged residues and 100 positively charged residues. The instability index of TGEV H16 S protein was 30.3, the aliphatic index was 90.97, and the grand average of hydropathicity was 0.035.

PEDV CV777 S protein included 1383 amino acids, and its molecular weight and isoelectric point were 151352.74 and 5.11, respectively. The protein contained 117 negatively charged residues and 85 positively charged residues. The instability index of PEDV CV777 S protein was 32.6, the aliphatic index was 93.21, and the grand average of hydropathicity was 0.123.

SADS-CoV GDS04 S protein contained 1130 amino acids, and its molecular weight and isoelectric point were 125996.51 and 6.46, respectively. The protein had 92 negatively charged residues and 87 positively charged residues. The instability index of SADS-CoV GDS04 S protein was 31.83, the aliphatic index was 84.77, and the grand average of hydropathicity was -0.029.

PDCoV HNZK-04 S protein encoded 1159 amino acids, and its molecular weight and isoelectric point were 128074.64 and 5.67, respectively. The protein contained 89 negatively charged residues and 73 positively charged residues. The instability index of PDCoV HNZK-04 S protein was 31.94, the aliphatic index was 93.96, and the grand average of hydropathicity was 0.027. Then, the system automatically generates a list of the physical and chemical properties of related proteins, and the analysis results are shown in Table 3.

### 3.2. Hydrophilicity and Hydrophobicity of Porcine Enteric Coronavirus Spike Proteins

**Figure 1:**
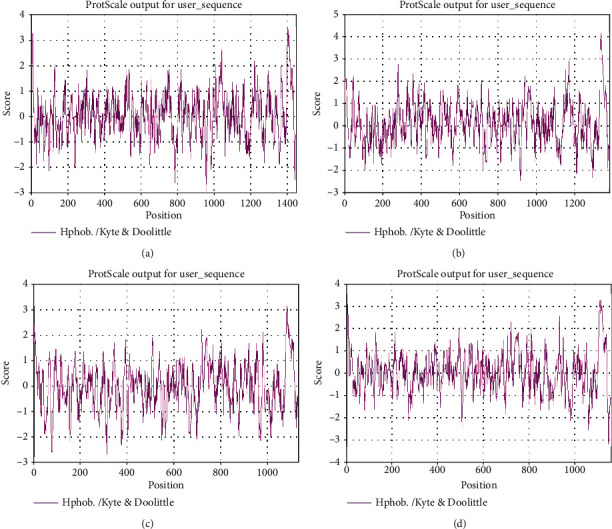
Hydrophilic and hydrophobic of porcine enteric coronavirus spike proteins: (a) TGEV H16 S protein, (b) PEDV CV777 S protein, (c) SADS-CoV GDS04 S protein, and (d) PDCoV HNZK-04 S protein. Note: the abscissa represents the amino acid position, and the ordinate represents the amino acid value. >0 is hydrophobic; <0 is hydrophilic.

The ExPASy-ProtScale software was used to analyze the amino acid sequences of porcine enteric coronavirus spike proteins for hydrophilicity and hydrophobicity, respectively. The asparagine (Asn) at positions 953 and 954 of TGEV H16 S protein had the strongest hydrophilic value of -2.967, and leucine (Leu) at position 1398 had the strongest hydrophobic value of 3.467 (Figure 1(a)). Asn at position 915 of PEDV CV777 S protein had the strongest hydrophilic value of -2.444, and Leu at position 1334 had the strongest hydrophobic value of 4.133 (Figure 1(b)). Asn at position 315 of SADS-CoV GDS04 S protein had the strongest hydrophilic value of -2.667, and Leu at position 1080 had the strongest hydrophobic value of 3.122 (Figure 1(c)). Asn at position 1145 of PDCoV HNZK-04 S protein had the strongest hydrophilic value of -3.189, and Leu at position 1112 had the strongest hydrophobic value of 3.244 (Figure 1(d)). These showed that porcine enteric coronavirus spike proteins were soluble proteins.

### 3.3. Transmembrane Region of Porcine Enteric Coronavirus Spike Proteins

**Figure 2:**
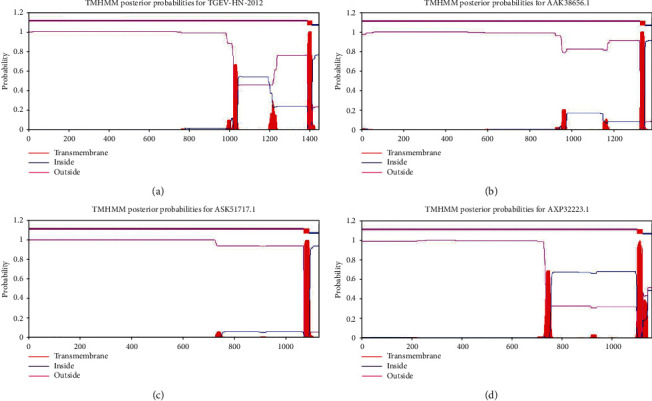
The prediction of transmembrane domain in porcine enteric coronavirus spike protein sequence with TMHMM: (a) TGEV H16 S protein, (b) PEDV CV777 S protein, (c) SADS-CoV GDS04 S protein, and (d) PDCoV HNZK-04 S protein.

The transmembrane regions of porcine enteric coronavirus spike protein amino acid sequences were predicted by TMHMM Server v.2.0. The results showed that the probability of amino acid 1 to1387 of TGEV H16 S protein was indicated with a purple line. The red area indicated that 1388 to 1410 amino acids could form a typical transmembrane helix region, and the blue area indicated 1411 to 1448 amino acids in virus M (Figure 2(a)). The probability of PEDV CV777 S protein 1 with 1324 amino acids was indicated with a purple line. The red area indicated that 1325 to 1347 amino acids could form a transmembrane spiral region, and the blue area indicated that 1348 to 1383 amino acids in virus M (Figure 2(b)). The prediction results showed that the probability of amino acids 1 to 1068 of SADS-CoV GDS04 S protein was indicated with a purple line. The red area indicated that 1069 to 1091 amino acids could form a typical transmembrane helix region, and the blue area indicated that 1092 to 1130 amino acids were in virus M (Figure 2(c)). The probability of PDCoV HNZK-04 S protein 1 with 1097 amino acids was indicated with a purple line. The red area indicated that 1098 to 1120 amino acids could form a transmembrane spiral region, and the blue area indicated 1121 to 1059 amino acids in virus M (Figure 2(d)). The prediction results showed that porcine enteric coronavirus spike proteins were mainly distributed on the outside of the virus envelope and could be used as membrane receptors for viruses to invade cells.

### 3.4. Signal Peptide of Porcine Enteric Coronavirus Spike Proteins

**Figure 3:**
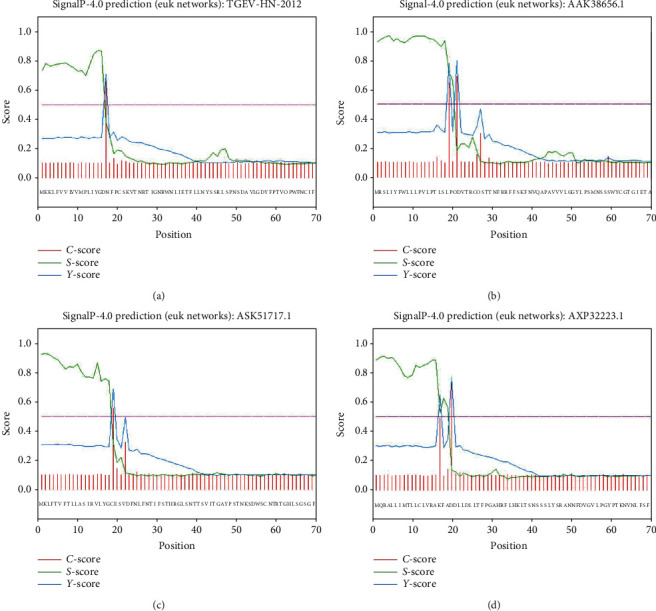
Analysis of signal peptide in the porcine enteric coronavirus spike proteins: (a) TGEV H16 S protein, (b) PEDV CV777 S protein, (c) SADS-CoV GDS04 S protein, and (d) PDCoV HNZK-04 S protein.

Signal peptide of porcine enteric coronavirus spike proteins was predicted by the Neural Network (NN) model of SignalP4.0 software. The results showed that there was a possible signal peptide in the range of residues 1-17 of the N-terminal of TGEV H16 S protein. The signal peptide sequence is MRSLIYFWLLLPVLPTLSLPQ. It was observed that the raw cleavage site score (*C* score) and the combined cleavage site score (*Y* score) both reached their peaks at the 17th place, while the signal peptide score (*S* score) began to decline at the 17th place. The splitting site was most likely to be located at the front of the maximum value of *Y* score, which was between amino acid 16 and amino acid 17 (LYG-DN) (Figure 3(a)).

There was a possible signal peptide in the range of residues 1-21 of the N-terminal of PEDV CV777 S protein. The signal peptide sequence is MRSLIYFWLLLPVLPTLSLPQ. It was observed that the raw cleavage site score (*C* score) and the combined cleavage site score (*Y* score) both reached their peaks at the 21st place, while the signal peptide score (*S* score) began to decline at the 21st place. The splitting site was most likely to be located at the front of the maximum value of *Y* score, which was between amino acid 20 and amino acid 21 (SLP-QD) (Figure 3(b)).

There was a possible signal peptide in the range of residues 1-20 of the N-terminal of SADS-CoV GDS04 S protein. The signal peptide sequence is MKLFTVFTLLASIRVLYGCE. It was observed that the raw cleavage site score (*C* score) and the combined cleavage site score (*Y* score) both reached their peaks at the 20th place, while the signal peptide score (*S* score) began to decline at the 20th place. The splitting site was most likely to be located at the front of the maximum value of *Y* score, which was between amino acid 18 and amino acid 19 (LYG-CE) (Figure 3(c)).

There may be a signal peptide in the range of residues 1-20 of the PDCoV HNZK-04 S protein. The signal peptide sequence is MQRALLIMTLLCLVRAKFAD. It could be seen that the raw cleavage site score (*C* score) and the combined cleavage site score (*Y* score) both reached their peaks at the 20th place, while the signal peptide score (*S* score) began to decline at the 20th place. The splitting site was most likely to be located at the front of the maximum value of *Y* score, which was between amino acid 19 and amino acid 20 (KFA-DD) (Figure 3(d)).

### 3.5. Phosphorylation Sites of Porcine Enteric Coronavirus Spike Proteins

**Figure 4:**
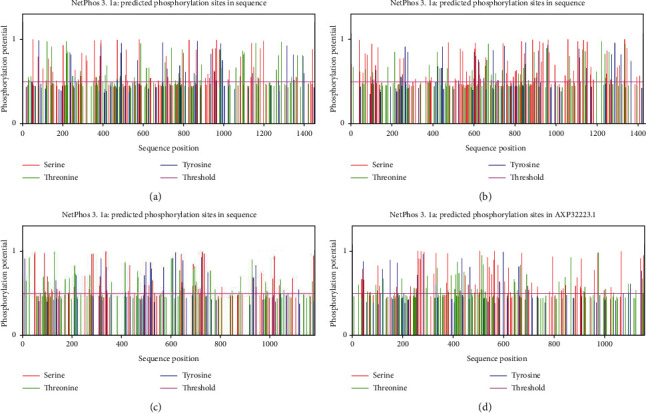
Analysis of phosphorylation sites of porcine enteric coronavirus spike proteins: (a) TGEV H16 S protein, (b) PEDV CV777 S protein, (c) SADS-CoV GDS04 S protein, and (d) PDCoV HNZK-04 S protein.

Almost all proteins undergo some chemical modifications during and after synthesis, such as the splicing of the peptide chain backbone and the side chains of specific amino acids. The phosphorylation of the protein is mainly carried out on tyrosine, serine, and threonine residues in the peptide chain. NetPhos 3.1 Server online software was used to predict the phosphorylation modification sites of porcine enteric coronavirus spike protein. The position with a score higher than 0.5 was the phosphorylation modification site. TGEV H16 S protein had 60 serine (Ser), 52 threonine (Thr), and 27 tyrosine (Tyr) modification sites (Figure 4(a)). PEDV CV777 S protein contained 80 serine (Ser), 39 threonine (Thr), and 24 tyrosine (Tyr) modification sites (Figure 4(b)). SADS-CoV GDS04 S protein had 52 serine (Ser), 20 threonine (Thr), and 37 tyrosine (Tyr) modification sites (Figure 4(c)). PDCoV HNZK-04 S protein contained 64 serine (Ser), 44 threonine (Thr), and 16 tyrosine (Tyr) modification sites (Figure 4(d)). The online software prediction was the same as the online database query result.

### 3.6. Glycosylation Sites of Porcine Enteric Coronavirus Spike Proteins

**Figure 5:**
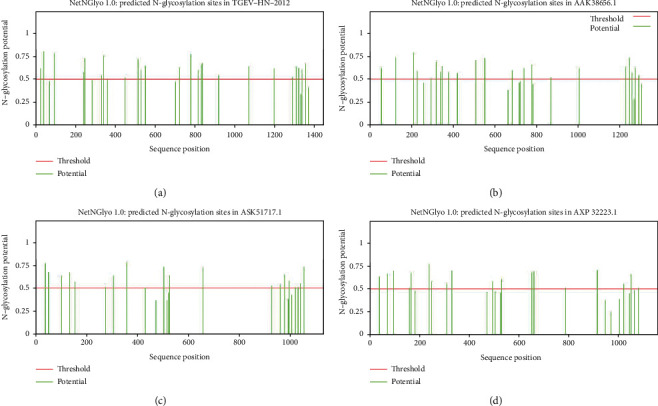
Analysis of glycosylation sites of porcine enteric coronavirus spike proteins: (a) TGEV H16 S protein, (b) PEDV CV777 S protein, (c) SADS-CoV GDS04 S protein, and (d) PDCoV HNZK-04 S protein.

Glycosylation modification can regulate protein functions, including N-linked and O-linked sugar chains. We used NetNGlyc/NetOGlyc 4.0 Server online software to predict N-type and O-type glycosylation modification sites for porcine enteric coronavirus spike protein. The prediction results showed that porcine enteric coronavirus spike proteins did not have O-glycosylation modification sites. TGEV H16 S protein contained 24 N-glycosylation sites (Figure 5(a)). PEDV CV777 S protein contained 22 N-glycosylation sites (Figure 5(b)). SADS-CoV GDS04 S protein contained 20 N-glycosylation sites (Figure 5(c)). PDCoV HNZK-04 S protein contained 18 N-glycosylation sites (Figure 5(d)). The specific glycosylation positions of porcine enteric coronavirus spike protein are shown in Table 4.

### 3.7. Epitopes of Porcine Enteric Coronavirus Spike Proteins

**Figure 6:**
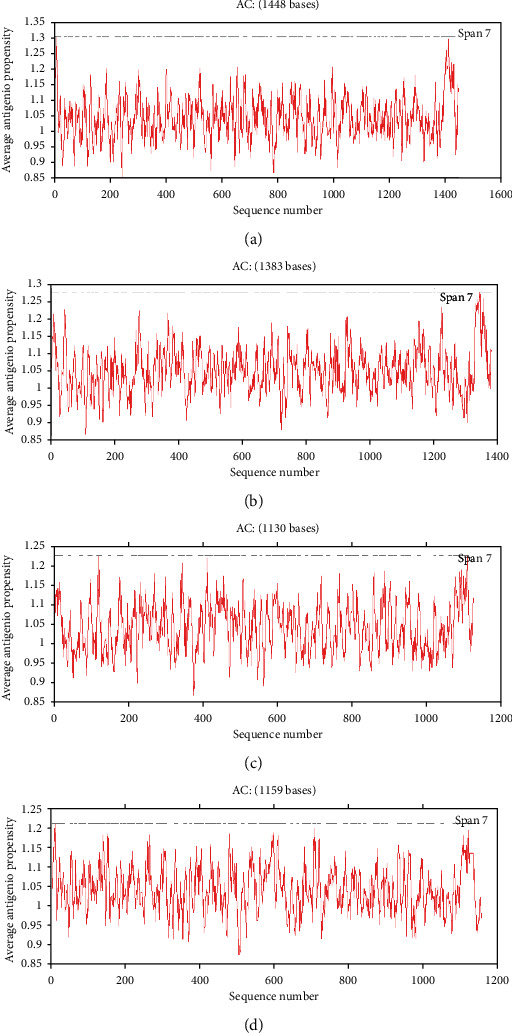
Analysis of epitope of porcine enteric coronavirus spike proteins: (a) TGEV H16 S protein, (b) PEDV CV777 S protein, (c) SADS-CoV GDS04 S protein, and (d) PDCoV HNZK-04 S protein.

The specificity of the S proteins depends on the type, nature, number, and spatial configuration of antigenic determinants. We used Predicting Antigenic Peptides online software to perform epitope prediction for porcine enteric coronavirus spike protein. The results showed that TGEV H16 S protein had 53 epitopes (Figure 6(a)). PEDV CV777 S protein had 51 epitopes (Figure 6(b)). SADS-CoV GDS04 S protein had 43 epitopes (Figure 6(c)). PDCoV HNZK-04 S protein had 52 epitopes (Figure 6(d)).

### 3.8. Structure Domain of Porcine Enteric Coronavirus Spike Proteins

**Figure 7:**
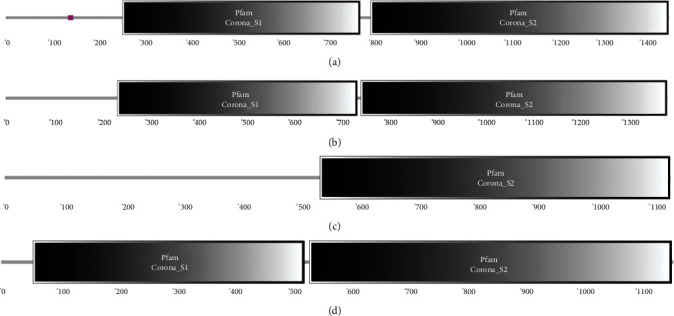
Structure domain of porcine enteric coronavirus spike proteins: (a) TGEV H16 S protein, (b) PEDV CV777 S protein, (c) SADS-CoV GDS04 S protein, and (d) PDCoV HNZK-04 S protein.

Different regions of the S proteins have different evolutionary rates, and some amino acids must be sufficiently conserved during the evolution process to achieve the corresponding biological functions. The functional regional subunit structure that could exist independently was the structure domain. Porcine enteric coronavirus S proteins were analyzed with the Simple Modular Architecture Research Tool (SMART). It was found that TGEV H16 S protein contained two typical functional domains, namely, spike_rec_binding between amino acids 330 and 583 and the highly conserved functional domain corona_S2 between amino acids 662 and 1266 (Figure 7(a)). PEDV CV777 S protein contained two functional domains, namely, spike_rec_binding between amino acids 330 and 583 and the highly conserved functional domain corona_S2 between amino acids 671 and 1270 (Figure 7(b)). It was found that SADS-CoV GDS04 S protein contained one typical functional domain, namely, the highly conserved functional domain corona_S2 between amino acids 535 and 1129 (Figure 7(c)). PDCoV HNZK-04 S protein also had two functional domains, namely, spike_rec_binding between amino acids 330 and 583 and the highly conserved functional domain corona_S2 between amino acids 671 and 1270 (Figure 7(d)).

### 3.9. Functional Motif of Porcine Enteric Coronavirus Spike Proteins

**Figure 8:**
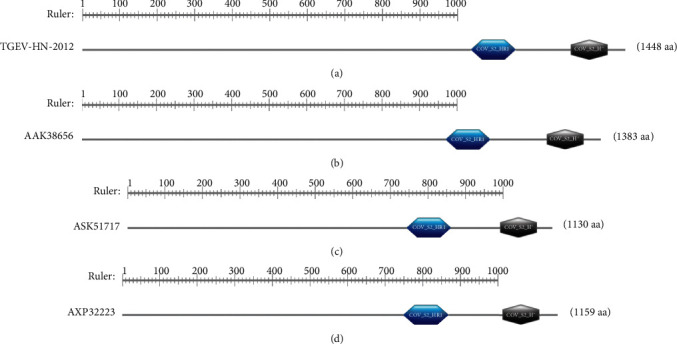
Functional motifs of porcine enteric coronavirus spike proteins: (a) TGEV H16 S protein, (b) PEDV CV777 S protein, (c) SADS-CoV GDS04 S protein, and (d) PDCoV HNZK-04 S protein.

The motif is a subunit in the structural domain, and its function is to reflect a variety of biological functions. According to the analysis of the PROSITE database, it was found that the functional motif of TGEV H16 S protein included S2-HR1 region profile of amino acids 1036-1155 and S2-HR2 region profile from amino acids 1304 to 1401 (Figure 8(a)). The functional motif of PEDV CV777 S protein included S2-HR1 region profile of amino acids 969-1088 and S2-HR2 region profile from amino acids 1240 to 1336 (Figure 8(b)). It was found that the functional motif of SADS-CoV GDS04 S protein included the S2-HR1 region profile of amino acids 750-855 and S2-HR2 region profile from amino acids 1001 to 1082 (Figure 8(c)). The functional motif of PDCoV HNZK-04 S protein included the S2-HR1 region profile of amino acids 750-869 and S2-HR2 region profile from amino acids 1013 to 1109 (Figure 8(d)).

## 4. Discussion

All coronaviruses have similarities in the genome composition and protein structure, including the structural proteins N, S, E, and M at the 3′-end and nonstructural proteins 1-16 (nsp1 to nsp16). The RBD in the S protein can bind to the corresponding receptor on the host cell membrane and then undergo membrane fusion with the host cell through S2 [6]. S protein can also induce the host's immune response and is a key protein for vaccine development. The change of S protein space structure directly affects the virulence of the virus. There are also differences in the tissue tropism and host range of the same genus of coronaviruses. Even TGEV H16 and PEDV CV777 and SADS-CoV GDS04 from the same genus do not necessarily use the same receptor [9, 13]. However, studies have shown that PDCoV HNZK-04 from the delta genus uses the same host receptor as TGEV, which belongs to the alpha genus [12].

The spatial structure of the protein determines its biological function. Changes in the spatial structure of the protein or chemical modification will affect its properties and functions. The prediction of function and structure of porcine enteric coronavirus S proteins using bioinformatics software will help to understand the mechanism of these proteins. The physicochemical properties of porcine enteric coronavirus S proteins were analyzed by ProtParam online software, which has reference value for the study of gene cloning and protein expression. The ExPASy-ProtScale software was used to analyze the hydrophilicity and hydrophobicity of porcine enteric coronavirus S proteins, and we found that the surfaces of these spike proteins were rich in hydrophilic amino acids. Generally, the hydrophobic region spanning the lipid bilayer is covalently bound to the fatty acid chain, and the hydrophilic polar part is located on the inner and outer surfaces of the membrane. TMHMM is a program that predicts the transmembrane helix based on the Markov model and combines the hydrophobicity of the transmembrane region, the length of the helix, the charge bias, and the topological limitation of membrane proteins. We predicted the transmembrane area of porcine enteric coronavirus S protein through the TMHMM Server v.2.0 online software. According to the prediction, we found that the S proteins of these four viruses all had a transmembrane region. The above prediction results were consistent with the information uploaded in the database.

Signal peptides mainly exist in secreted proteins, transmembrane proteins, and eukaryotic organelles, which can promote the secretion of proteins outside the cell. The position of the porcine enteric coronavirus S protein signal peptide was predicted with SignalP4.0. The prediction results showed that TGEV H16 S protein, PEDV CV777 S protein, SADS-CoV GDS04 S protein, and PDCoV HNZK-04 S protein contained signal peptide at residues 1-17, residues 1-21, residues 1-20, and residues 1-20, respectively.

Phosphorylation can mediate protein activity and enhance the ability of protein interaction. Through the analysis of NetPhos 3.1 Server online software, it was found that TGEV H16 S protein, PEDV CV777 S protein, SADS-CoV GDS04 S protein, and PDCoV HNZK-04 S protein contained 139, 143, 109, and 124 phosphorylation modification sites, which belong to phosphoproteins, respectively. The specific glycosylation modification is differently labeling the corresponding glycosylation sites of the spike protein, which can change the conformation of the polypeptide in the spike protein, thereby increasing the stability and regulating function. The N-type and O-type glycosylation modification sites were predicted for TGEV H16 S protein, PEDV CV777 S protein, SADS-CoV GDS04 S protein, and PDCoV HNZK-04 S protein through NetNGlyc4.0Serve and NetOGlyc4.0Serve online software, respectively. The results showed that these four viruses did not contain O-glycosylation modification sites, and all contained many N-glycosylation modification sites. Predicting Antigenic Peptides online software was used to predict the epitope of porcine enteric coronavirus S protein. Predicting Antigenic Peptides online software, which was based on the method of Kolas-kar and Tongaonkar, was used to predict the epitope of porcine enteric coronavirus S proteins. The prediction results showed that TGEV H16 S protein, PEDV CV777 S protein, SADS-CoV GDS04 S protein, and PDCoV HNZK-04 S protein had 53, 51, 43, and 52 epitopes, respectively.

According to SMART and PROSITE database analysis, we found that S proteins of TGEV H16, PEDV CV777, and PDCoV HNZK-04 all have two functional domains, spike_rec_binding and corona_S2. spike_rec_binding functional domain contained two main functional motifs, NTD and CTD, and the corona_S2 functional domain contained the functional motifs of HR1 and HR2. SADS-CoV GDS04 S protein contained only one functional domain, the corona_S2 domain. Studies have shown that CTD is often used as a key location for binding to receptors [14]. It can be seen that the epitope in CTD deserves more attention. For porcine enteric coronavirus, the design of a vaccine will search for epitopes on the spike protein of the virus. Then, the corresponding antigen epitope is transferred into the expression system by genetic engineering, which can produce the corresponding antigen protein. The four S genes were compared with multiple sequences, and we found that the sequence similarities were less than 60%. The sequence similarities between TGEV H16 S gene and PEDV CV777 S gene, SADS-CoV GDS04 S gene, and PDCoV HNZK-04 S gene were 58.1%, 52.1%, and 57.1%, respectively.

Due to the different standards and parameters adopted by each software, different bioinformatics software will have different prediction results. In addition, with the continuous updating and improvement of protein databases, the results of using bioinformatics software to predict biological functions at different times will also change. To obtain credible prediction results, it is necessary to use a variety of software to analyze at the same time. This experiment analyzes the biological function of S protein to provide a reference for the later study of coronavirus and its potential functions. At the same time, the study of the biological and immune properties of the spike protein is beneficial to the use of the S protein as a target in the process of vaccine design. It is also meaningful to clarify whether the coronavirus currently circulating in animals poses a potential threat to humans.

## Figures and Tables

**Table 1:** The information of S gene sequences of coronaviruses.

Strain name	Genome source	Genome accession	Position in genome	Protein accession	PDB ID
TGEV	H16	FJ755618	20365-24711	ACN71196	—
PEDV	CV777	AF353511	20638-24789	AAK38656	6U7K
SADS	GDS04	MF167434	20476-23868	ASK51717	6M39
PDCoV	HNZK-04	MH708124	19324-22803	AXP32223	—

**Table 2:** Bioinformatics software-related information.

Name of software	URL	Function
ProtParam	https://web.expasy.org/protparam/	Analyze the physical and chemical properties of protein
ExPASy-ProtScale	https://web.expasy.org/protscale/	Analysis of protein affinity and hydrophobicity
TMHMM Server v.2.0	https://www.cbs.dtu.dk/services/TMHMM/	Transmembrane analysis of proteins
SignalP4.0	https://www.cbs.dtu.dk/services/SignaIP-4.0/	Predictive signal peptide
NetPhos 3.1 Server	https://www.cbs.dtu.dk/services/NetPhos/	Prediction of phosphorylation site
NetNGlyc4.0Serve	https://www.cbs.dtu.dk/services/NetNGlyc/	Prediction of N-type glycosylation sites
Predicting Antigenic Peptides	http://imed.med.ucm.es/Tools/antigenic.pl	Prediction of protein epitopes
SMART	http://smart.embl-heidelberg.de/	Protein domain analysis tool
PROSITE	http://prosite.expasy.org/	Prediction of functional motif
Clustal Omega	https://www.ebi.ac.uk/Tools/msa/clustalo/	Multiple sequence alignment

**Table 3:** Physical and chemical properties of porcine enteric coronavirus spike proteins.

Characteristic	TGEV-S protein	PEDV-S protein	SADS-S protein	PDCoV-S protein
Number of amino acids	1448	1383	1130	1159
Formula	C_7155_H_11401_N_18_	C_6786_H_10483_N_1751_	C_5641_H_8655_N_149_	C_5685_H_8923_N_1511_O_1_
	_61_O_2169_S_64_	O_2060_S_56_	_5_O_1662_S_62_	_746_S_53_
Molecular weight	159888.38	151352.74	125996.51	128074.64
Theoretical (PI)	5.26	5.11	6.46	5.67
Number of negatively charged residues	126	117	92	89
Number of positively charged residues	100	85	87	73
Instability index (II)	30.30	32.60	31.83	31.94
Aliphatic index	90.97	93.21	84.77	93.96
Grand average of hydropathicity	0.035	0.123	-0.029	0.027

**Table 4:** N-glycosylation sites of porcine enteric coronavirus spike protein sequence.

TGEV-S protein		PEDV-S protein		SADS-S protein		PDCoV-S protein	
Position	Seq	Position	Seq	Position	Seq	Position	Seq
26-29	NRTI	57-60	NSSS	39-42	NTTS	41-44	NSSS
42.45	NYSS	127-130	NKTL	52-55	NKSD	73-76	NSTL
94-97	NITS	213-216	NVTS	104-107	NLTW	98-101	NHTL
243-246	NGTT	230-231	NCTG	135-138	NSTS	161-164	NYTT
250-253	NCTD	397-300	NHTM	154-157	NGSY	168-171	NSTF
334-337	NNTV	321-324	NDTS	275-278	NMSA	240-243	NLTD
345-348	NFTK	341-344	NLSF	305-308	NYTT	250-253	NTTI
449-552	NLTT	348-351	NSSD	358-361	NRTI	310-313	NISA
516-519	NKSV	378-381	NSTV	429-432	NFTF	330-333	NTSY
532-535	NITI	422-425	NFTG	503-506	NGTA	493-496	NNTV
554-557	NITL	511-514	NITV	525-528	NCTN	530-533	NCTK
725-728	NVSD	553-556	NVTN	654-657	NVSQ	651-654	NITN
780-783	NYTN	685-688	NVTS	924-927	NNTI	660-663	NFSS
818-821	NVTH	740-743	NCTE	957-960	NLSY	787-790	NISL
833-835	NVTI	778-781	NISI	973-976	NLTF	913-916	NGTH
839-842	NFTI	870-873	NFTN	992-995	NKTF	1019-1022	NQTV
920-923	NSSE	1006-1009	NITS	1016-1019	NMSS	1048-1051	NLTV
1073-1076	NITQ	1229-1132	NLTS	1026-1029	NLTA	1078-1081	NNTL
1199-1202	NGTH	1246-1249	NKTL	1035-1038	NISA		
1293-1296	NATV	1258-1261	NRTG	1049-1052	NVSN		
1310-1313	NQTV	1275-1278	NLTG				
1323-1326	NWTV	1292-1295	NTTE				
1340-1343	NLTG						
1357-1360	NTTV						

## Data Availability

Databases used are from https://web.expasy.org/protparam/, https://web.expasy.org/protscale/, http://www.cbs.dtu.dk/services/TMHMM/, http://www.cbs.dtu.dk/services/NetPhos/http://imed.med.ucm.es/Tools/antigenic.pl, http://smart.embl-heidelberg.de/http://prosite.expasy.org/, and https://www.ebi.ac.uk/Tools/msa/clustalo/.
